# Association Between Trait Empathy and Resting Brain Activity in Women With Primary Dysmenorrhea During the Pain and Pain-Free Phases

**DOI:** 10.3389/fpsyt.2020.608928

**Published:** 2020-11-26

**Authors:** Wanghuan Dun, Tongtong Fan, Qiming Wang, Ke Wang, Jing Yang, Hui Li, Jixin Liu, Hongjuan Liu

**Affiliations:** ^1^Department of Intensive Care Unit, First Affiliated Hospital of Xi'an Jiaotong University, Xi'an, China; ^2^Department of Rehabilitation Medicine, First Affiliated Hospital of Xi'an Jiaotong University, Xi'an, China; ^3^Department of Medical Imaging, First Affiliated Hospital of Xi'an Jiaotong University, Xi'an, China; ^4^School of Life Science and Technology, Center for Brain Imaging, Xidian University, Xi'an, China

**Keywords:** empathy, primary dysmenorrhea, interpersonal reactivity index, partial least squares, functional MRI

## Abstract

Empathy refers to the ability to understand someone else's emotions and fluctuates with the current state in healthy individuals. However, little is known about the neural network of empathy in clinical populations at different pain states. The current study aimed to examine the effects of long-term pain on empathy-related networks and whether empathy varied at different pain states by studying primary dysmenorrhea (PDM) patients. Multivariate partial least squares was employed in 46 PDM women and 46 healthy controls (HC) during periovulatory, luteal, and menstruation phases. We identified neural networks associated with different aspects of empathy in both groups. Part of the obtained empathy-related network in PDM exhibited a similar activity compared with HC, including the right anterior insula and other regions, whereas others have an opposite activity in PDM, including the inferior frontal gyrus and right inferior parietal lobule. These results indicated an abnormal regulation to empathy in PDM. Furthermore, there was no difference in empathy association patterns in PDM between the pain and pain-free states. This study suggested that long-term pain experience may lead to an abnormal function of the brain network for empathy processing that did not vary with the pain or pain-free state across the menstrual cycle.

## Significance

This study reveals the association of resting brain activity and different trait empathy scores, and suggests that repeated menstrual pain had an effect on empathy processing that did not vary with the pain or pain-free states. It provides valuable insights into the neural mechanisms of empathy in primary dysmenorrhea.

## Introduction

Empathy is a complex psychological construct that enable the sharing of the emotions, pain, and sensations of others, and also allow the exertion of cognitive ability in our interactions (1). The capacity to resonate with others may be influenced by many factors, such as motivational factors (2), gender differences (3), or current states (4). The ability to understand the pain in fellow human beings may be inhibited when individuals experience anger or depressed emotions (5). However, mechanisms of empathy in chronic pain are still unclear and have not received scientific attention compared with those related with healthy individuals.

Neuroimaging has been developed to assess the neural mechanisms of empathy in healthy individuals, and has consistently identified a core network comprising the anterior insula (AI) and the mid-cingulate cortex (MCC) in empathic states (6). Given that empathy can fluctuate in character in different states, a limited number of neuroimaging studies assessed empathy with subjects in different states. An electroencephalographic (EEG) study showed that the motor component of empathy for pain can be influenced by the mood states (5). Mira and colleagues reported that participants who were exposed to pressure pain yielded a lower activity in AI and the aMCC (7). Additionally, functional magnetic resonance imaging (fMRI) on subjects with congenital insensitivity to pain suggested that the empathy for pain in patients was regulated by different mechanics (8). Given that long-term pain can lead to maladaptive alteration in the central nervous system, including regions associated with empathy (9), whether empathy in clinical populations with pain can be influenced by prior pain experiences is still unknown. Primary dysmenorrhea (PDM), a very common type of cyclic menstrual pain without organic causes in female adolescents, induces recurrent, spontaneous, painful and pain-free states (10). Women with PDM have a poorer mood state during menstruation than preovulatory phases (11). Hence, PDM can be used as a great clinical model to study both the effects of long-term menstrual pain on empathy and the difference of the empathy between painful and pain-free states.

The interpersonal reactivity index (IRI) is the most common psychometric tool used to measure an individual's empathy. It uses four subscales including perspective taking, fantasy, empathic concern, and personal distress (12). Despite the findings of prior neuroimaging studies that examined the relationship between the brain and empathy based on IRI, all of them employed the mass-univariate general linear models (GLM) (1). In addition to the assumptions that must be met, mass-univariate tests increase the occurrence of false positive findings owing to multiple comparisons, and thus attenuate minor effects caused by corrections (13).

In the current study, our main aims were to investigate: (1) whether or not the empathy related to the resting brain activity could be affected by long-term pain, and (2) whether or not there were differences in empathy between painful and pain-free phases in women with PDM. Resting-state functional data and clinical variables were collected from 46 PDM women and 46 healthy controls (HC) during the periovulatory, luteal, and menstruation phases. We used the multivariate analysis method partial least squares (PLS) correlation to explore the relationship between patients' trait empathy and brain resting activity at three time points.

## Materials and Methods

### Subjects

In the present study, we recruited 57 right-handed PDM patients and 53 age- and education-matched healthy participants from a local university with advertisements. All the subjects were diagnosed by a gynecologist and took part in conventional MRI scans to exclude pelvic or anatomical brain abnormalities. The following are inclusion criteria for PDMs: (1) menstrual cycle regularity of approximately 27–32 days, (2) menstrual pain during the past 6 months, (3) averaged pain intensity score >4 on a visual analog scale ((0 = no pain, 10 = worst imaginable pain) during the past 6 months. The inclusion criteria for HCs was similar to PDMs but without menstrual pain. Subject exclusion criteria were: (1) psychological or neurologic disease, (3) organic pelvic disease, (4) smoking, alcohol, or drug abuse, (5) childbirth or plan for pregnancy, (6) a history of other comorbid chronic pain conditions (e.g., irritable bowel syndrome, fibromyalgia, etc.), (7) use of oral contraceptives, hormonal supplements, Chinese medication, or the intake of central-acting medication during the last 6 months before MRI, or (8) any MRI contraindications. Resting-state functional (fMRI) data and various pathopsychological data were collected during the periovulatory, luteal, and menstruation phases. Urine kits were used to quantify luteinizing hormone to verify the periovulatory phase of participants. Eight PDMs and five HCs were excluded for missing fMRI data. Three patients and two healthy individuals lost their questionnaire information. Thus, 46 PDMs and 46 HCs were used in subsequent analyses.

Informed consent was obtained from all the participants and the experimental procedures were explained. The Institutional Review Board of the First Affiliated Hospital of the Medical College Xi'an Jiaotong University approved the study that complied with the Declaration of Helsinki.

### Trait Empathy Assessment

The Chinese version of the IRI was used in this study. It is a self-report questionnaire designed to assess participants' trait empathy. It consists of four subscales, each of which measures a separate aspect of empathy: perspective taking (PT), fantasy scale (FS), empathic concern (EC), and personal distress (PD). PT refers to the ability to spontaneously adopt the perspectives of other people and see things from their points-of-view, FS refers to the tendency to transpose themselves into fictional situations, EC refers to a tendency to experience feelings of compassion and concern for the observed individual, and PD is self-oriented and measures feelings of anxiety and distress that result from witnessing another person's negative experience (14).

Each subscale contains seven items and participants rate the degree to which each item describes them on a five-point Likert scale ranging from 0 (“Does not describe me well”) to 4 (“Describes me very well”). For each subscale, a total score is calculated with higher scores indicating a higher functional level at the aspect of empathy.

### MRI Data Acquisition

MRI was conducted at the Xi'an Jiaotong University during the menstruation, luteal, and periovulation phases, using a 3 T scanner (GE Signa HDxt, Milwaukee, WI, USA) with an eight-channel phased array head coil. The participants were told to close their eyes during the scans, but were instructed to remain awake and not to think about anything in particular. We obtained high-resolution three-dimensional (3D) longitudinal relaxation (T1) structural images with an axial fast spoiled gradient recalled sequence (FSGR) with the following parameters: voxel size = 1 mm^3^, repetition time (TR)/echo time (TE) = 1,900 ms/2.6 ms, field-of-view (FOV) = 256 × 256 mm, acquisition matrix = 256 × 256, flip angle = 12°, and slices = 140. Resting-state functional images were obtained using transverse relaxation star (T2^*^)-weighted, single-shot gradient-recalled echo-planar-imaging (EPI) sequence (TR = 2,000 ms, TE = 30 ms, matrix size = 64 × 64, slices = 35, slice thickness = 4.0 mm, FOV = 240 × 240 mm, flip angle = 90°.

### MRI Data Preprocessing

Imaging data were analyzed using the FSL and Automated Functional Neuro-Imaging (http://afni.nimh.nih.gov/afni) software. Scripts containing the processing commands used in the current study have been released as part of the 1,000 Functional Connectome Project (http://www.nitrc.org/projects/fcon_1000) (15). A standard data preprocessing strategy was performed. Specifically, (1) the first five EPI volumes of each subject were discarded to allow subjects to become familiar with the scanning environment and eliminate magnetization variations to the steady state, and (2) the remaining images were corrected for slice timing, 3D head motion, time series despiking effects, and spatial smoothing and four-dimensional (4D) mean-based intensity normalization; (3) the time series from each voxel in the corrected images was temporally filtered with a bandpass filter (0.01–0.08 Hz) that eliminated the linear and quadratic trends. (4) The remaining images were then spatially normalized to the Montreal Neurological Institute 152 space and resampled to 2 mm isotropic voxels, and (5) eight nuisance signals (WM, cerebrospinal fluid signals and 24 motion parameters) were regressed out.

## MRI Data Analysis

### Regional Homogeneity (ReHo)

ReHo is a data-driven method evaluated using Kendall's coefficient of concordance (KCC) (16). It characterizes the local functional connectivity (FC) between a given voxel and its nearest neighboring voxels, and can integrate regional homogeneity in both structure and function. A previous study reported that ReHo obtained highly reliable (test-retest) results in a resting-state fMRI data analysis (17). Thus, ReHo is regarded as a robust and crucial measure of brain activity (18).

We used the resting-state fMRI data analysis toolkit (http://www.restfmri.net/forum/index.php) to evaluate the ReHo values in our fMRI datasets. First, ReHo maps of each subject were generated by computing the KCC of the time series of each voxel with its nearest 26 neighboring voxels. To eliminate the influence of individual variability, individual KCC images among each voxel were divided by their own mean KCC values estimated within a whole-brain mask for standardization. Finally, a Gaussian kernel (full-width at half-maximum: 4 mm) was used to smooth the standardized ReHo images to reduce noise and residual differences.

### Statistical Analysis on Demographic and Clinical Data

Statistical tests were performed using SPSS Statistics 20.0 (SPSS Inc, Chicago, IL). Two-sample *t*-test was used to evaluate the differences in demographic characteristics (age, education, menstrual cycle, etc.) and clinical information (VAS, IRI) between HC and PDM. We then applied repeated measures analysis of variance (ANOVA) to test for a significant difference between clinical information within the group and the interaction effect between groups during the three phases. Normality of the data was examined and confirmed prior to statistical analysis. Results were considered significant at *p* < 0.05.

### Statistical Analysis of MRI Data Using PLS Correlation Analysis

Behavior PLSC was used to explore resting brain activity associated with different dimensions of empathy. Behavior variables for each individual are entered into one matrix (behavior matrix), and resting brain activity in each voxel across the brains of each individual was expressed by another matrix (brain matrix). Herein, we included the four behavior variables: PT, FS, EC, and PD.

The first step is to compute the cross-covariance between behavior and brain matrix (19). The cross-covariance matrix was then subjected to singular value decomposition (SVD). A set of orthogonal latent variables (LVs) was generated and represented the maximal covariance between brain and behavioral measures (20). LVs consisted of d (the singular value that characterize the brain-behavior correlation strength), brain saliences (weightings across voxels that best express the contributions of every voxel to the brain-behavior correlation explained by this LV), and behavior saliences (weights across behavioral variables that indicate the intensity of the contribution of each behavioral variable to the brain-behavior correlation explained by this LV). The number of LVs is equal to the rank of the number of behavioral variables multiplied by the number of groups (20). Therefore, the PLS analysis in our study produced eight LVs, as we have two groups and four behavioral variables.

Additionally, we calculated brain scores for each participant as the dot product of the resting brain activity and the brain saliences used to quantify the contribution of each individual to each LV. Brain scores are the projections of every participant's brain data onto the salient brain pattern. Large absolute values of brain scores represent a strong contribution to the pattern and the scores closer to zero demonstrate a weaker contribution.

To determine the statistical significance of each LV in the PLS correlation analysis, we applied permutation testing with the use of 5,000 permutations. These reordered randomly the rows of the data matrix and maintained the behavior matrix unchanged. The LVs with *p* < 0.05 were considered significant so that the LVs were generalizable to the population. In the last step, we repeated bootstrap resampling 5,000 times to assess reliability of the effect in each voxel. Bootstrap ratios (BSRs: original saliences/bootstrap standard errors) were then obtained. The absolute values of saliences with BSR thresholds >3 were considered reliable, and were equivalent to *p*-values of approximately 0.01 (21). Otherwise, this involves random sampling (with replacement) of the brain and behavior matrices.

Subsequently, we obtained the distribution of the Pearson's correlation coefficient among the brain scores and each level of trait empathy in the two groups at each period. Finally, two-sample *t*-tests were performed between groups during the three phases.

## Results

### Demographic Data and Clinical Characteristics

Forty-six right-handed patients with PDM and forty-six gender-matched HCs were recruited in the study. There was no significant difference between PDM patients and HCs in age, years of education, menstrual cycle, and age at menarche (*p* > 0.05). In this study, all PDM patients had a long history of menstrual pain (10.48 ± 0.25 years, Table 1).

**Table 1:** Demographics of the studied participants.

**Characteristic**	**HC (*n* = 44) mean (SE)**	**PDM (*n* = 44) mean (SE)**	***t***	***p***
Age (years)	24.11 (0.21)	23.93 (0.34)	0.46	0.647
Education (years)	17.45 (0.15)	17.61 (0.23)	−0.57	0.568
Menstrual cycle (days)	29.14 (0.23)	29.34 (0.27)	−0.57	0.568
Age of menarche (years)	12.48 (0.14)	12.57 (0.17)	−0.42	0.674
History of menstrual pain (years)	—	10.48 (0.25)	—	—
Duration of menstrual pain (days)	—	1.94 (0.14)	—	—

*PDM, primary dysmenorrhea; HC, healthy controls; SE, standard error. Comparisons of the subjects' basic information between PDM and HC groups were conducted with two-sample t-tests (p < 0.05 was considered significant)*.

PDM patients had significantly higher VAS scores than HCs during the periovulatory, luteal, and menstruation phases (Table 2). Additionally, repeated measures ANOVA test demonstrated that VAS scores yielded significant group-time interactions and significant differences within the PDM group at the three time points. For IRI subscales, two-sample *t*-tests yielded significant group differences with respect to the FS both in the periovulatory and menstruation phases, and empathic concern in the periovulatory phase (*p* < 0.05). No significant differences were found between groups and group-time interactions at perspective taking and personal distress.

**Table 2:** Clinical comparison at periovulatory, luteal, and menstruation phases.

**Behavior assessment**		**Time 1 mean (SE)**	**Time 2 mean (SE)**	**Time 3 mean (SE)**	***p*^a^**	***p*^b^**
VAS	HC	0.07 (0.03)	0.31 (0.14)	0.47 (0.14)	0.056	0.000^*^
	PDM	1.07 (0.31)	1.30 (0.31)	4.27 (0.31)	0.000^*^	
	*p*^c^	0.002^*^	0.005^*^	0.000^*^		
Perspective taking	HC	11.18 (0.53)	10.61 (0.51)	10.82 (0.56)	0.426	0.93
	PDM	11.52 (0.59)	10.95 (0.47)	10.93 (0.56)	0.264	
	*p*^c^	0.666	0.626	0.887		
Fantasy scale	HC	13.89 (0.61)	14.86 (0.57)	14.25 (0.53)	0.083	0.15
	PDM	16.25 (0.56)	16.00 (0.58)	16.16 (0.65)	0.833	
	*p*^c^	0.004^*^	0.159	0.020^*^		
Empathic concern	HC	16.45 (0.54)	16.84 (0.45)	15.98 (0.42)	0.196	0.30
	PDM	18.00 (0.41)	17.39 (0.46)	16.86 (0.49)	0.074	
	*p*^c^	0.024^*^	0.400	0.173		
Personal distress	HC	7.36 (0.53)	7.43 (0.55)	7.32 (0.60)	0.969	0.61
	PDM	8.16 (0.54)	8.68 (0.59)	8.59 (0.66)	0.502	
	*p*^c^	0.296	0.126	0.157		

*PDM, primary dysmenorrhea; HC, healthy controls; SE, standard error. Repeated measurements ANOVA*:

a *comparison of data at different times within the group*;

b comparison of interactions (Group*Times) between groups at different times. Two-sample t-test:

c Comparison of differences between groups at the same period

(*: *p < 0.05)*.

### Association Patterns Between Resting Brain and Empathy

**Figure 1:**
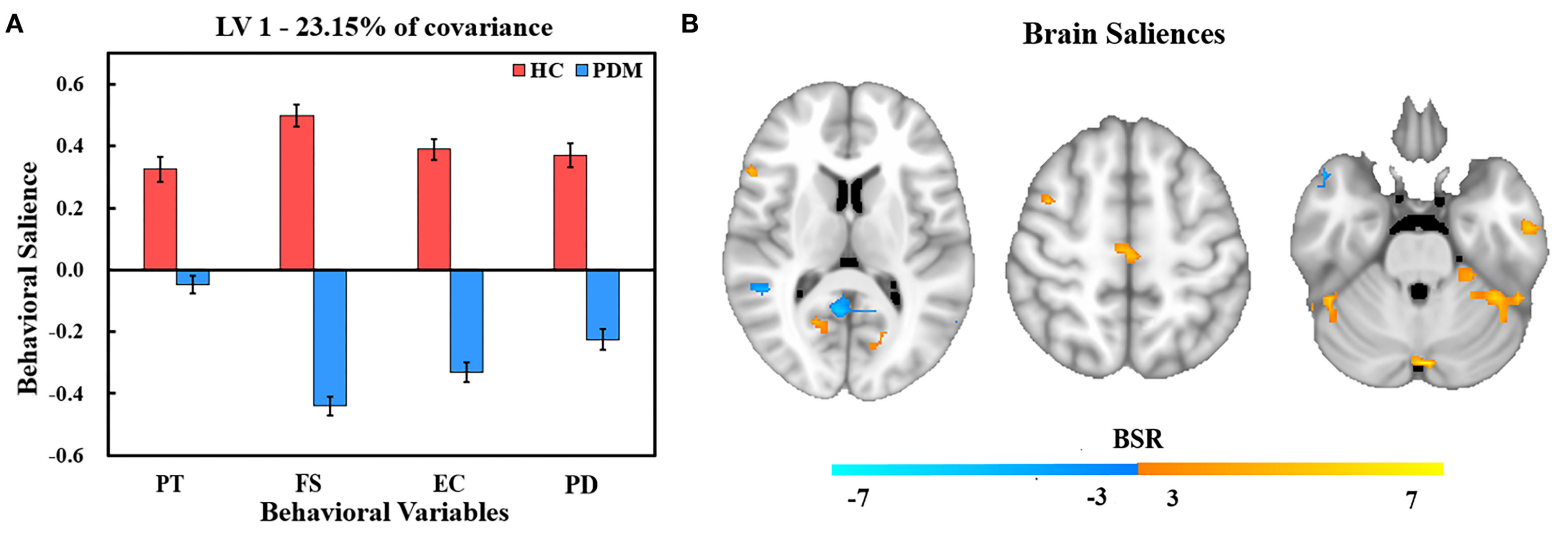
PLS correlation analysis results of LV 1. **(A)** Bar graph of behavioral salience with stability estimated using the bootstrap method in the two studied groups. **(B)** Resting brain activity showing brain regions in which variations were associated with trait empathy. The map is generated with the threshold set at *p* = 0.01 (BSR = ±3). In the HC group, warm regions indicated a positive correlation between resting brain activity, empathy scores, and cool regions showed a negative correlation between the resting brain activity and empathy scores. In contrast, the association was reversed in the PDM group (warm, negative correlation; cool, positive correlation). PT, perspective taking; FS, fantasy; EC, empathic concern; PD, personal distress.

**Figure 2:**
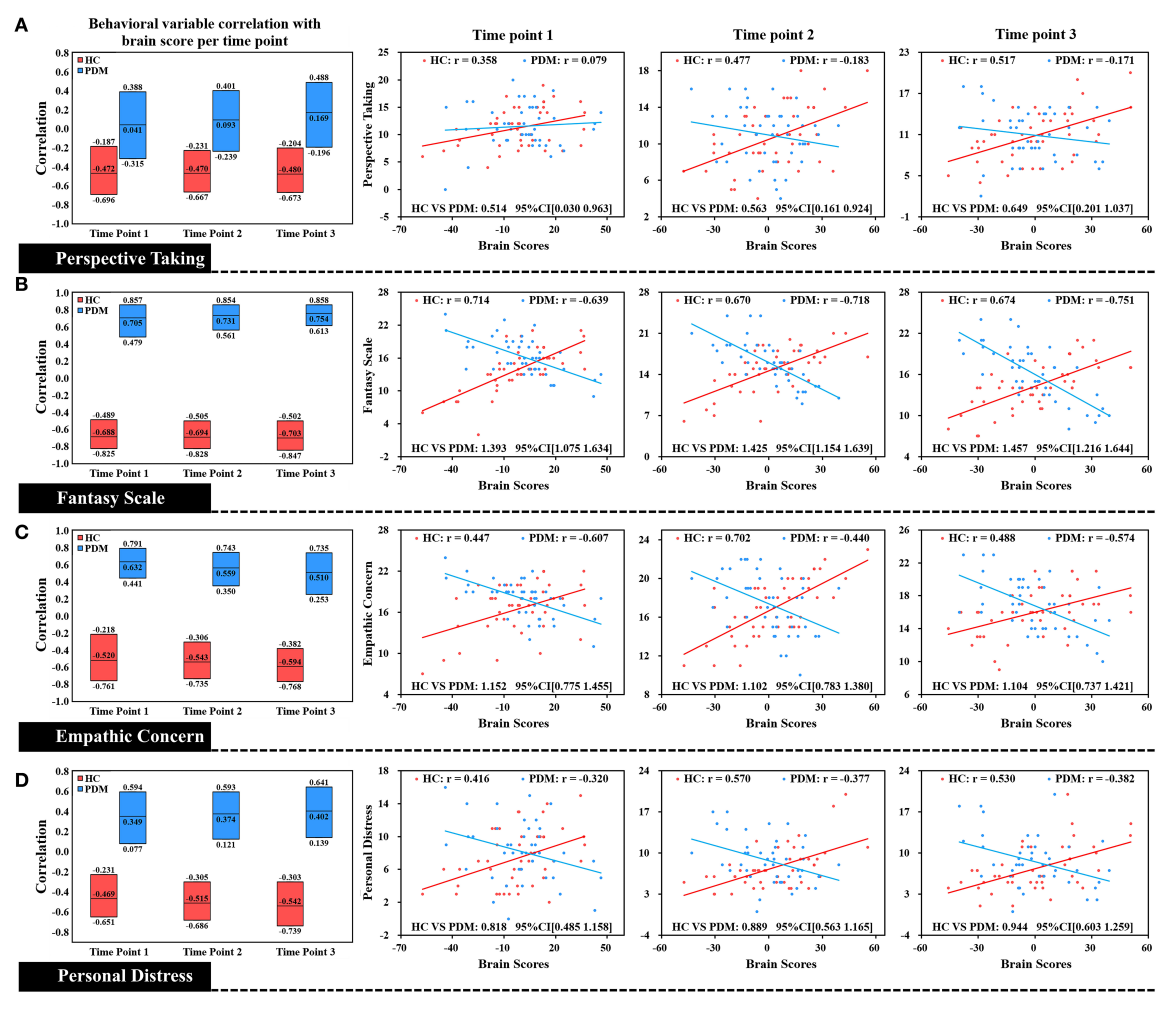
Correlation of each level of trait empathy with brain scores of LV 1. In the box plot, the middle line of the box was the original correlation between empathy and brain score in the same phase, and the boundary of the box was the 95% CI of the trait empathy correlation with brain scores based on the use of 5,000 bootstraps. The original correlation between empathy and brain scores is shown by the scatter plot. There was a significant difference between HC and PDM in different dimensions of trait empathy in correlation with brain scores at the periovulatory, luteal, and menstruation phases through the effect-size analysis. There were no significant correlation changes in both groups during the three phases. HC, red; PDM, blue.

The PLS analysis generated 10 latent variables (LVs), but only the first significant LV (LV1) and the second significant LV (LV2) were statistically significant in relation to trait empathy and corresponding resting brain patterns in PDM patients and HCs. LV1 accounted for 23.15% of the shared covariance between clinical and resting brain measures and identified abnormal regulation brain patterns to trait empathy in PDM women compared with HCs (Figure 1). LV2 accounted for 19.31% covariance and identified similar regulation patterns between groups (Figure 2).

### Abnormal Regulation Brain Patterns to Trait Empathy in PDM Women Compared With HCs

Figure 1A shows behavior salience characteristics corresponding to the first significant LV for PLS correlation analysis. In HCs group, FS was the strongest contributor to LV1, followed by EC, PD, and PT. Different dimensions of trait empathy have similar contributions to the brain-behavior correlation in PDM patients with the highest behavior salience in FS, followed by EC, PD, and PT. This indicates that all dimensions of trait empathy contributed to the brain-behavior correlation. However, the correlation patterns for behaviors and resting brain activities of the HC and PDM groups were different. This indicates an abnormal regulation pattern to empathy by brain regions in PDM women compared with HCs.

The brain regions that showed an association with trait empathy identified in LV1 are shown in Figure 1B. Trait empathy was correlated with both decreased and increased regional resting brain activity. In the HC group, we found that the resting brain activity was negatively correlated with trait empathy in the middle temporal gyrus, superior temporal gyrus, right lingual gyrus, middle occipital gyrus, posterior cingulate, right inferior parietal lobule (IPL), postcentral gyrus, and left supramarginal gyrus (Table 3). Positive associations between trait empathy and resting brain activity were in the right inferior temporal gyrus, left middle frontal gyrus, left fusiform gyrus, left precuneus, superior frontal gyrus, inferior frontal gyrus (IFG), and right supplementary motor area. The brain regions that correlated with trait empathy in PDM patients were abnormal. This identified opposite associations with trait empathy in these regions.

**Table 3:** The brain regions that have reliability contribution to behavior-brain covariance in LV 1.

**Region of activation**	**Side**	**Sizes**	**Peak coordinates**			
			**x**	**y**	**z**	**BSRs**
Inferior Temporal Gyrus	R	54	48	−48	−30	5.705
Middle Temporal Gyrus	L	54	−39	18	39	6.626
Middle Temporal Gyrus	R	20	48	−45	12	−6.453
Superior Temporal Gyrus	R	21	48	12	−21	−6.112
Lingual Gyrus	R	24	3	−87	−18	−5.071
Middle Occipital Gyrus	R	25	48	−69	−12	−4.785
Posterior Cingulate	R	58	12	−57	12	−5.854
Fusiform Gyrus	L	24	−54	−12	−30	5.422
Inferior Parietal Lobule	R	22	57	−33	36	−5.101
Precuneus	L	52	−12	−48	39	5.654
Superior Frontal Gyrus	L	37	−15	6	72	6.043
Inferior Frontal Gyrus	R	31	60	18	15	5.536
supplementary motor area	R	31	3	−21	60	5.967
Postcentral Gyrus	R	16	12	−51	72	−6.159
Supramarginal Gyrus	L	24	−54	−42	15	−5.818

*BSRs, Original saliences/bootstrap standard errors; R, right hemisphere; L, left hemisphere; Peak coordinates refer to the MNI space; MNI, Montreal Neurological Institute*.

Correlation plots of brain scores with different dimensions of trait empathy are shown in Figure 2. In the HC group, brain scores are positively correlated with all dimensions of trait empathy in the periovulatory, luteal, and menstruation phases. In contrast to the HCs, there is a negative correlation in the PDM group regardless of the moderate positive correlation with PT in the periovulatory phase. Brain score correlations at each level of empathy yielded significant differences between HC and PDM in the periovulatory, luteal, and menstruation phases (none of the bootstrapped 95% confidence intervals of the correlation coefficients shown in Figure 2 crosses zero). Moreover, significant changes of the correlation in both groups did not occur in the three phases.

### Similar Brain Regulation Patterns to Trait Empathy Between PDM Women and HCs

**Figure 3:**
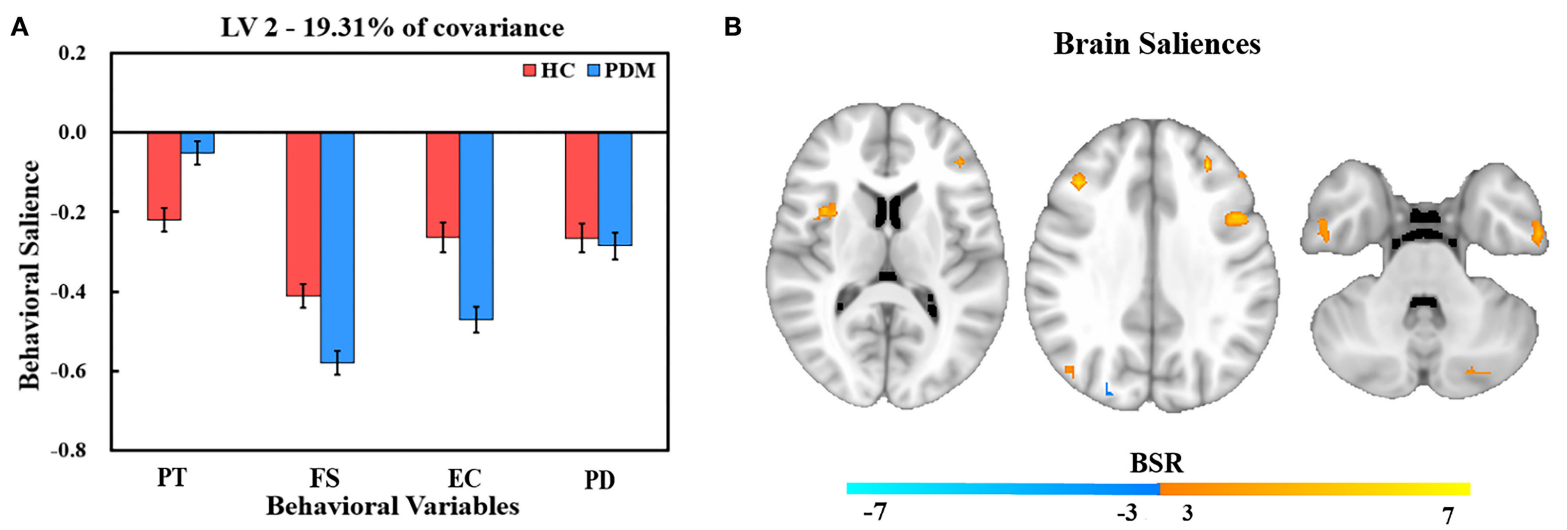
PLS correlation analysis results of LV 2. **(A)** Bar graph of behavioral salience with stability estimated based on the use of bootstraps in the two groups. **(B)** Resting brain activity showing brain regions in which variations were associated with trait empathy. The map is the threshold at the equivalent value of *p* = 0.01 (BSR = ±3). Owing to the negative salience in both groups, warm regions indicated a negative correlation between resting brain activity and empathy, while cool regions showed a positive correlation between resting brain activity and empathy.

Behavior salience contributing to LV2 are shown in Figure 3A. In comparison with LV1, LV2 reflects a similar regulation pattern of brain to trait empathy between PDM women and HCs. We found that FS was the strongest contributor to LV2, followed by PD, EC, and PT in HCs. The highest behavior salience was also found in FS, followed by EC, PD, and PT in the PDM group.

The correlation of resting brain and trait empathy between the groups in LV2 was similar (Figure 3B). There was a significantly negative association in brain regions in both groups that involved the inferior temporal gyrus, right middle temporal gyrus, lingual gyrus, right anterior insula, middle frontal gyrus, IFG, and left IPL (Table 4). Positive association between trait empathy and resting brain activity were found in the left superior temporal gyrus, left inferior occipital gyrus, right posterior cingulate, right precuneus, and middle frontal gyrus.

**Table 4:** Brain regions with reliability contributions to the behavioral brain covariance in LV 2.

**Region of activation**	**Side**	**Sizes**	**Peak coordinates**			
			**x**	**y**	**z**	**BSRs**
Inferior Temporal Gyrus	R	22	63	−15	−30	5.477
Inferior Temporal Gyrus	L	20	−54	−12	−36	5.782
Middle Temporal Gyrus	R	28	−42	−78	15	4.868
Superior Temporal Gyrus	L	20	−42	3	−24	−5.983
Lingual Gyrus	R	28	18	−90	−3	4.448
Inferior Occipital Gyrus	L	21	−42	−75	−9	−4.724
Posterior Cingulate	R	17	6	−42	3	−4.924
Insula	R	21	39	9	9	5.4
Precuneus	R	29	27	−87	27	−4.9459
Middle Frontal Gyrus	R	85	42	27	33	6.849
Middle Frontal Gyrus	L	51	−45	33	24	6.521
Middle Frontal Gyrus	R	42	36	6	57	−6.736
Inferior Frontal Gyrus	L	20	−42	9	30	6.191
Inferior Parietal Lobule	L	16	−42	−51	51	5.119

*BSRs, Original saliences/bootstrap standard errors; R, right hemisphere; L, left hemisphere Peak coordinates refer to the MNI space; MNI, Montreal Neurological Institute*.

**Figure 4:**
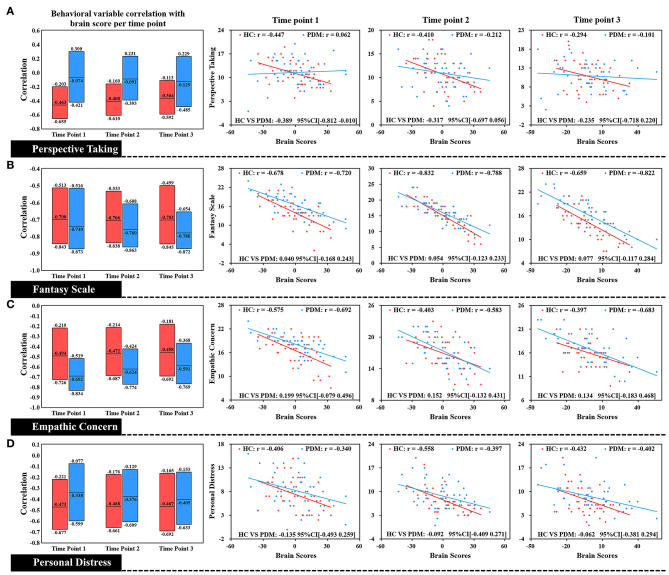
Correlation of each level of trait empathy with brain scores of LV 2. In the box plot, the middle line of the box is the original correlation between empathy and brain score in the same phase. The boundary of the box was the 95% CI of the trait empathy correlation with brain scores based on the use of 5,000 bootstraps. The original correlation between empathy and brain score is shown by the scatter plot. There were no significant differences between HC and PDM in the EC, FS, and PD correlations with brain scores at the periovulatory, luteal, and menstruation phases based on effect-size analysis. The correlations in both groups did not significantly change during the three phases.

Brain scores correlated with different dimensions of trait empathy in three phases in both groups is shown in Figure 4. We found that brain scores were negatively correlated with FS, EC, and PD, in the three phases in the HC group. There was also a negative correlation in the PDM group of the brain scores with all dimensions of trait empathy. Specifically, no significant difference between HC and PDM was found in the correlations of the brain scores with EC, FS, and PD, based on effect-size analysis (all of the bootstrapped 95% confidence intervals of the correlation coefficients shown in Figure 4 cross zero). Similar to LV1, the correlation in both groups did not significantly change during the three phases.

## Discussion

In this study, it was shown based on the use of multivariate PLS correlation analyses that part of the identified empathy-related brain network in the PDM yielded an opposite activity compared with HC that indicated an abnormal regulation pattern to empathy in PDM. Additionally, these abnormal networks in PDM did not change across the menstrual cycle. This suggested that long-term menstrual pain may affect different aspects of trait empathy regardless of the pain or pain-free phases.

### Trait Empathy-Related Brain Functional Networks in HCs

In our findings, we found that a set of brain regions were involved in different dimensions of trait empathy in HCs. These brain regions mainly included the right anterior insula, IFG, IPL, middle and superior temporal gyri, posterior cingulate, and precuneus. Functional neuroimaging studies on the neural mechanisms of empathy highlighted the AI, IFG, and IPL as parts of common networks in empathy for pain (22). Neuropsychological data provided further evidence that the lesions that involved the AI (23) and IFG (24) were associated with impairments of empathy. Additionally, the posterior cingulate, precuneus, and temporal gyri were also reported in research of empathy (25). With respect to the subscale of IRI, in a gray matter study of trait levels of empathy, Banissy et al. revealed that affective empathy scores on the PD and EC was linked with reduced gray matter volume in the insula, and EC scores were negatively related to gray matter volume both in the left precuneus and the left IFG (1). Levels of gray matter volume in the temporal gyrus were reported to be associated with participant PT and EC scores (26). Our data that related brain activity in the insula, IFG, temporal gyrus, and precuneus to empathy concern abilities were therefore in line with the studies of structural correlates of empathy. It is necessary to note that empathy is a multifaceted construct and consists of an emotional system and a cognitive system (27). The present theories propose that empathy relies strongly on the involvement of various neural networks (22), and is mediated by different series of interaction regions (28). Based on the multivariable analysis of PLS correlation, our study was consistent with the above, and further suggested a comprehensive interaction network related to different aspects of empathy.

### Brain Dysfunction Networks Associated With Empathy in PDM Women

In our results, a brain network that had an opposite regulation pattern to empathy as compared with the HC group was found in the PDM, and mainly involved the posterior cingulate, precuneus, IPL and IFG. PCC and precuneus are considered as parts of the default mode network (DMN) that has contributed immensely to various cognitive processing and has generated tremendous scientific attention (29, 30). Abnormal function of DMN have been found in a broad range of pain disorders as well as significant correlations between DMN and current clinical pain. Besides, alterations of DMN function indicate brain reorganization due to chronic pain (31). As DMN is recruited in attention related events (32), disengagement of attention to pain, such as mind wandering, is of benefit to individuals to cope with pain, and has an effect on the activity of the DMN network (33). In our study, it is likely that women with prolonged menstrual pain have a tendency to raise their attention to pain that leads to abnormal activities in the PCC and the precuneus. On the other hand, neuroimaging studies have indicated that the DMN networks involved in self-referential processing (34, 35). Specifically, empathy is inextricably associated with self-referential cognition (36). This suggested that empathy relied on self-referential processing to infer and represent the feelings of others (37). Moreover, dynamic causal modeling combined with canonical variance analysis revealed that functional integration within the DMN is associated with self-reported empathy (38). The DMN network plays a prominent role in empathy-related processing, and altered function may be linked with aberrant empathy. Our findings may indicate that long-term menstrual pain entails changes of activity and structure in PCC and precuneus that leads to changes of empathic processing.

The IPL and IFG are the two components of the newly defined frontoparietal network. Previous studies have reported abnormalities of frontoparietal network in chronic pain conditions (39). There is also converging evidence that this network was associated with endogenous pain modulation. For instance, Kong et al. indicated that functional connectivity of the frontoparietal network was involved in the prediction of the modulation of pain (40). Using a pattern-based regression method, Wager et al. mentioned that the anticipatory activity of the frontoparietal network reliably contributed to the prediction of placebo analgesia response (41). It is plausible to hypothesize that the alert structure of the IPL and IFG may reflect a dysfunctional pain modulation response. In addition, the frontoparietal network plays a critical role in the circuit of imitation and action representation that modulates and shapes emotional processing to impact empathy processing along with superior temporal sulcus (42). In this circuit, the IPL receives visual description of the action from superior temporal sulcus and code for precise kinesthetic properties, and then sends the information to IFG mirror neurons to code the goal of the action. The IFG sends copies of the motor plans back to superior temporal sulcus (43). Furthermore, a neuroimaging study has identified shared representation in empathy for pain (44). It is conceivable that the network underlying empathy partially overlaps with pain-related network (including IFG and IPL), and persistent pain can cause dysregulation of the empathy pathway by affecting the overlapping network.

Another interesting finding was that AI, middle, and superior temporal gyri, and the inferior occipital gyrus had similar activities compared with HC. There is accumulating evidence supporting the fact that the insula cortex plays an important role in the mediation of pain perception (45). Chronic pain studies have indicated that the morphometric abnormalities of the insula have been mentioned in chronic low-back pain studies (46). However, our results did not find any abnormal activity of AI in PDM women. The human insula has been divided into several subdivisions, including the ventral anterior insula (vAI) and dorsal anterior insula (dAI) (47), each of which has an influence on cognitive and affective processing (48). Recently, a meta-analysis that examined gray matter (GM) changes indicated that chronic pain led to increased GM in the dAI but decreased GM in the vAI (49). As noted, chronic pain alters both the cognitive and emotional domains (50). Accordingly, the increase in dAI may be equal to a decrease in vAI and represents normal activity in the entire AI in chronic pain. In our research, the empathic processing ability and intrinsic structure of AI may be disrupted by menstrual pain, but the overall activity appears to be normal. These findings provide novel insights into the impact of chronic pain on brain processing of empathy.

The underlying functional role of the temporal and inferior occipital gyri in chronic pain is not clear. Hence, the reason for which the temporal and inferior occipital gyri maintain similar activities in relation with empathy in PDM is difficult to explain. We have to consider the possibility that these regions were not affected by long-term menstrual pain and operated normally in empathic processing. There is another possibility that the intensity of menstrual pain is not high enough to affect the regulation of the temporal and inferior occipital gyri in empathy. To reach a full understanding of this issue, more research is needed on the empathy of pain disorders.

### Changing Patterns of the Relationship Between Brain Regions and Empathy During the Menstrual Pain

In our results, the correlation of each aspect of empathy trait with brain scores yielded significant differences between PDM and HC during the periovulatory, luteal, and menstruation phases. As it was reported, prolonged nociceptive stimulus induced a form of adaptive reorganization and maladaptive plasticity in the brain (51). Neuroimaging studies with positron emission tomography and voxel-based morphometry disclosed state-related brain changes in metabolism and morphology in the various pain states (52, 53). There were also findings that indicated trait-related abnormal structure and functional connectivity during pain-free phases without menstrual pain (51, 54, 55). Our results showed significantly different FS and EC scores as well as correlation of empathy with brain scores between two groups in periovulatory phases that suggested that repeated menstrual pain might have an effect on different levels of empathy in the painful and in the pain-free phases. Additionally, there were no differences in the correlation patterns to empathy in pain and pain-free phases. This indicated that the aberrant trait empathy in PDM does not vary with the pain or pain-free state throughout the menstrual cycle.

## Data Availability Statement

The raw data supporting the conclusions of this article will be made available by the authors, without undue reservation.

## Ethics Statement

The studies involving human participants were reviewed and approved by The Institutional Review Board of the First Affiliated Hospital of the Medical College Xi'an Jiaotong University. The patients/participants provided their written informed consent to participate in this study. Written informed consent was obtained from the individual(s) for the publication of any potentially identifiable images or data included in this article.

## Author Contributions

HLiu and JL conceived the idea of this study and conducted the experiment. WD and TF detailedly analyzed results and writing the paper. QW helped with the data analysis and processing. KW, JY, and HLi contributed to the collection of data and samples. All authors revised the manuscript.

## Conflict of Interest

The authors declare that the research was conducted in the absence of any commercial or financial relationships that could be construed as a potential conflict of interest.
